# Chemotherapy is of prognostic significance to metaplastic breast cancer

**DOI:** 10.1038/s41598-024-51627-1

**Published:** 2024-01-12

**Authors:** Meilin Zhang, Jingjing Yuan, Maoli Wang, Mingdi Zhang, Hongliang Chen

**Affiliations:** Department of breast surgery, Obstetrics and Gynecology Hospital of Fudan University, Shanghai, 200011 China

**Keywords:** Cancer, Breast cancer, Cancer therapy, Cancer

## Abstract

This study aimed to evaluate the significance of chemotherapy (CT) among metaplastic breast cancer (MpBC), and to compare the survival outcomes between triple negative MpBC (MpBC-TNBC) and triple negative invasive ductal carcinoma (IDC-TNBC). SEER database was indexed to identify female unilateral primary MpBC diagnosed from 2010 to 2017. Patients were classified into neoadjuvant chemotherapy (NAC) with response (NAC-response), NAC-no response, adjuvant chemotherapy, and no CT. Breast cancer-specific survival (BCSS) and overall survival (OS) was estimated using the Kaplan–Meier method and compared by log-rank test. Cox regression was used to evaluate the independent prognostic factors. A 1:4 propensity score matching method was adopted to balance baseline differences. Altogether 1186 MpBC patients were enrolled, among them 181 received NAC, 647 received adjuvant CT and 358 did not receive any CT. Chemotherapy was an independent favorable prognostic factor. NAC-response and adjuvant CT had a significant or an obvious trend of survival improvement compared with NAC-no response or no CT. MpBC-TNBC was an independent unfavorable prognostic factor compared with IDC-TNBC. Among them, there was significant or trend of survival improvement among all TNBCs receiving NAC or adjuvant CT compared with no CT. Chemotherapy was of important significance to MpBC prognosis and should be integrated in comprehensive treatment for MpBC.

## Introduction

Metaplastic breast cancer (MpBC) is a rare histologic subtype of breast cancers, which accounts for 0.2–1% of invasive breast carcinoma^1–3^. It has a more aggressive clinical course and poorer survival outcomes compared with invasive ductal carcinoma (IDC)^4,5^. Two recent studies of the National Cancer Database (NCDB) reported that MpBC was the histologic subtype associated with the worst overall survival^1,2^.

Due to its rarity and heterogeneity, there are currently no standard treatment strategies for MpBC^6–8^. Many researches demonstrated poor response to chemotherapy in MpBC, especially low pCR rate in neoadjuvant chemotherapy (NAC). In their opinion, a radical surgery is of first priority for MpBC and the significance of chemotherapy (CT) is under doubt. However, as MpBC generally presents with higher histologic grade, tumor stage, Ki-67 index, and with triple negative (TN) phenotype, chemotherapy, both neoadjuvant and adjuvant, is still considered essential. As a result, there is still much controversy in the significance of chemotherapy for the improvement of survival outcomes for MpBC. Optimizing systemic therapy options is considered a priority for managing MpBC in clinical practice.

Furthermore, MpBC most commonly shows a TN phenotype (MpBC-TNBC). Contradictory results exist whether this histology of MpBC is correlated with a significantly poorer prognosis compared with classical triple negative IDC (IDC-TNBC). The use of chemotherapy in MpBC is mostly extrapolated from clinical trial results involving typical IDC. Although MpBC is believed to be chemoresistance to some extent, the survival differences between MpBC-TNBC and IDC-TNBC based on chemotherapy response are still unknown.

This study aimed to evaluate the significance of chemotherapy among MpBC, and to compare the survival outcomes between MpBC-TNBC and IDC-TNBC.

## Results

### Baseline characteristics among metaplastic carcinoma of the breast receiving chemotherapy (neoadjuvant or adjuvant) or not

Altogether 1186 patients with MpBC were enrolled based on the inclusion criteria between 2010 and 2017. Among them, there were 1023 cases with no special type of MpBC (MpBC-NST) and 163 cases with definite subgroup of MpBC (31 with spindle cell carcinoma, 46 with squamous cell carcinoma, 50 with low-grade adenosquamous carcinoma, 19 with sarcomatoid carcinoma, four with chondroid differentiation and 13 with fibromatosis).

Median age of the 1186 MpBC cases was 61 years old (22–100 years old). The majority of them had histologic grade III disease (978 patients, 82.5%) and TN subtype (844 patients, 71.2%). There were 303 patients (25.5%) in stage I, 725 patients (61.1%) in stage II and 158 patients (13.3%) in stage III. Most patients (828 cases, 69.8%) received chemotherapy, among whom, 181 patients (15.3%) received NAC and 647 patients (54.6%) received adjuvant CT. Only 358 patients (30.2%) did not receive any CT. Among patients receiving NAC, 22 cases (12.2%) achieved CR, 67 cases (37.0%) achieved PR, 48 cases (26.5%) achieved CR or PR and 44 cases (24.3%) showed no response to NAC. A higher proportion of older patients, grade I-II and N0 disease were observed among patients who did not receive CT. On the contrary, patients who received NAC had a higher proportion of T4 and N2-3 disease. Patients who underwent NAC or adjuvant CT were more likely to receive radiation therapy (69.6% in NAC and 54.1% in adjuvant CT). The clinical-pathological characteristics were summarized in Table 1.

**Table 1:** Clinical-pathological characteristics of MpBC among (neo) adjuvant CT or no CT groups.

	Neoadjuvant CT		Adjuvant CT		No CT		P
	No	%	No	%	No	%	
Year of diagnosis							
2010–2011	34	18.8%	140	21.6%	93	26.0%	0.286
2012–2013	38	21.0%	157	24.3%	82	22.9%	
2014–2015	44	24.3%	161	24.9%	87	24.3%	
2016–2017	65	35.9%	189	29.2%	96	26.8%	
Age							
≤ 60	124	68.5%	354	54.7%	86	24.0%	< 0.001
> 60	57	31.5%	293	45.3%	272	76.0%	
Race							
White	131	72.4%	486	75.1%	299	83.5%	0.009
Black	35	19.3%	112	17.3%	36	10.1%	
Others*	15	8.3%	49	7.6%	23	6.4%	
Histology							
Others	22	12.2%	67	10.4%	74	20.7%	< 0.001
NST	159	87.8%	580	89.6%	284	79.3%	
Histologic grade							
I	1	0.6%	18	2.8%	35	9.8%	< 0.001
II	20	11.0%	74	11.4%	60	16.8%	
III	160	88.4%	555	85.8%	263	73.5%	
Stage							
I	7	3.9%	181	28.0%	115	32.1%	< 0.001
IIA	60	33.1%	306	47.3%	146	40.8%	
IIB	48	26.5%	102	15.8%	63	17.6%	
IIIA	31	17.1%	33	5.1%	11	3.1%	
IIIB	28	15.5%	21	3.2%	17	4.7%	
IIIC	7	3.9%	4	0.6%	6	1.7%	
T							
T1	13	7.2%	196	30.3%	120	33.5%	< 0.001
T2	84	46.4%	362	56.0%	159	44.4%	
T3	54	29.8%	68	10.5%	60	16.8%	
T4a–c	26	14.4%	20	3.1%	17	4.7%	
T4d	4	2.2%	1	0.2%	2	0.6%	
N							
N0	101	55.8%	529	81.8%	324	90.5%	< 0.001
N1	53	29.3%	95	14.7%	22	6.1%	
N2	20	11.0%	19	2.9%	6	1.7%	
N3	7	3.9%	4	0.6%	6	1.7%	
Surgery							
BCS	52	28.7%	331	51.2%	161	45.0%	< 0.001
Mastectomy	129	71.3%	316	48.8%	197	55.0%	
Radiation therapy							
Yes	126	69.6%	350	54.1%	120	33.5%	< 0.001
No or unknown	55	30.4%	297	45.9%	238	66.5%	
ER							
Positive	51	28.2%	119	18.4%	60	16.8%	0.004
Negative	130	71.8%	528	81.6%	298	83.2%	0.004
PR							
Positive	31	17.1%	76	11.7%	40	11.2%	0.107
Negative	150	82.9%	571	88.3%	318	88.8%	
HER2							
Positive	17	9.4%	40	6.2%	11	3.1%	0.009
Negative	164	90.6%	607	93.8%	347	96.9%	
Subtype							
HR + /HER2–	52	28.7%	147	22.7%	75	20.9%	0.003
HR + /HER2 +	8	4.4%	11	1.7%	2	0.6%	
HR–/HER2 +	9	5.0%	29	4.5%	9	2.5%	
TNBC	112	61.9%	460	71.1%	272	76.0%	
Response to NAC							
CR	22	12.2%	–	–	–	–	
PR	67	37.0%	–	–	–	–	
CR or PR	48	26.5%	–	–	–	–	
No response	44	24.3%	–	–	–	–	

*American Indian/AK native, Ascian/Pacific Islander.

### Factors associated with chemotherapy among MpBC patients

As the significance of chemotherapy for MpBC was still somewhat controversial, the factors associated chemotherapy among MpBC were then explored. Variables with statistically significant difference (P < 0.05) in the one-way logistic regression associated with chemotherapy were younger age, non-white race, higher histologic grade or stage and radiation therapy. Based on the multivariate logistic regression model, age less than 60 years, histologic grade II–III, stage II–III and radiation therapy were independently correlated with chemotherapy (Table 2) (Hosmer Lemeshow P = 0.123).

**Table 2:** Multivariate logistic regression of factors associated with chemotherapy among MpBC.

Factors	One-way logistic regression			Multivariate logistic regression		
	OR	95% CI	P	OR	95% CI	P
Year of diagnosis	1.052	0.997–1.109	0.064			
Age						
≤ 60y vs. > 60y	4.319	3.268–5.709	< 0.001	4.100	3.052–5.509	< 0.001
Race			0.002			0.237
Black vs. white	1.979	1.340–2.922	0.001	1.423	0.930–2.180	0.104
Others* vs. white	1.348	0.821–2.215	0.238	1.202	0.695–2.077	0.510
Histologic grade			< 0.001			0.001
II vs. I	2.886	1.153–5.504	0.001	3.361	1.658–6.815	0.001
III vs. I	5.008	2.815–8.910	< 0.001	4.685	2.462–8.913	< 0.001
Stage			0.001			0.014
II vs. I	1.510	1.139–2.003	0.004	1.539	1.114–2.126	0.009
III vs. I	2.231	1.430–3.480	< 0.001	1.792	1.099–2.921	0.019
Radiation therapy						
Yes vs. no or unknown	2.682	2.070–3.475	< 0.001	2.710	2.041–3.599	< 0.001
Surgery						
BCS vs. mastectomy	1.053	0.821–1.351	0.684			
Subtype			0.031			0.323
HR–/HER2 + vs. TNBC	2.008	0.957–4.211	0.065	1.596	0.725–3.515	0.246
HR + /HER2 + vs. TNBC	4.517	1.045–19.533	0.044	3.136	0.666–14.772	0.148
HR + /HER2– vs. TNBC	1.262	0.933–1.707	0.132	1.099	0.785–1.538	0.582

*American Indian/AK native, Ascian/Pacific Islander.

Hosmer and Lemeshow P = 0.123.

### Survival outcomes stratified by chemotherapy in MpBC

**Figure 1:**
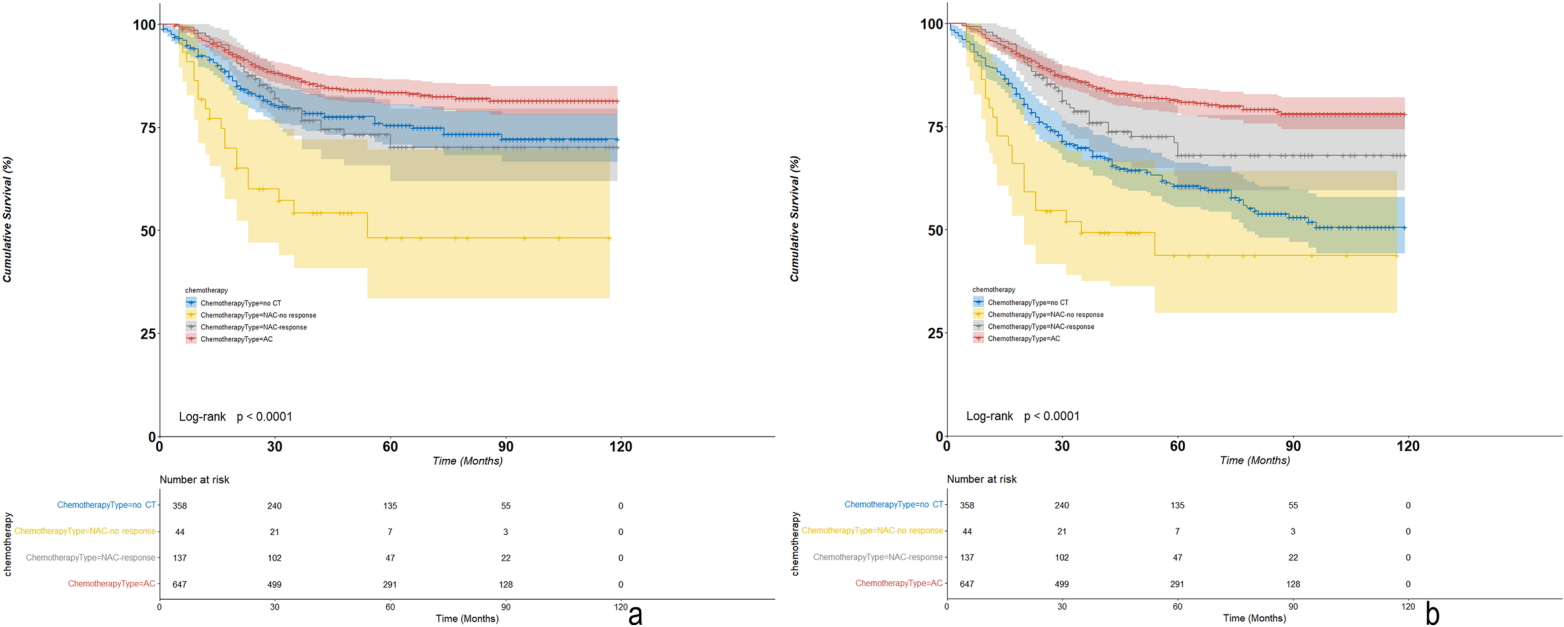
Kaplan–Meier survival curves of BCSS and OS among MpBC stratified by chemotherapy types ((**a**) KM curves of BCSS; (**b**) KM curves of OS).

After a median follow-up of 48 months (1–119 months), 321 MpBC patients died, among whom, 239 patients died due to breast cancer. There were statistically significant differences in BCSS and OS among MpBC patients with NAC-response, NAC-non response, adjuvant CT or without CT (P < 0.001) (Fig. 1). According to COX multivariate analysis, chemotherapy was the independent prognostic factor for both BCSS (P = 0.009) and OS (P < 0.001). Compared with no CT, NAC-response and adjuvant CT had a significant or an obvious trend of survival improvement (no CT as reference, HR for NAC-response was 0.691 (0.444–1.077) for BCSS and 0.479 (0.321–0.715) for OS; HR for adjuvant CT was 0.658 (0.480–0.902) for BCSS and 0.451 (0.346–0.587) for OS), while NAC-no response did not improve survival outcomes compared with no CT (no CT as reference, HR for NAC-no response was 1.266 (0.744–2.155) for BCSS and 0.984 (0.610–1.587) for OS) (Table 3).

**Table 3:** Multivariate COX regression of independent prognostic factors for MpBC.

	BCSS				OS			
	Univariate regression		Multivariate regression		Univariate regression		Multivariate regression	
	HR (95% CI)	P	HR (95% CI)	P	HR (95% CI)	P	HR (95% CI)	P
Year of diagnosis	0.956 (0.902–1.013)	0.125			0.965 (0.917–1.015)	0.166		
Age								
> 60y	Ref.		Ref.		Ref.		Ref.	
≤ 60y	0.748 (0.579–0.966)	0.026	0.762 (0.577–1.005)	0.054	0.527 (0.419–0.664)	< 0.001	0.606 (0.472–0.777)	< 0.001
Race		0.716				0.716		
White	Ref.	0.495			Ref.			
Black	1.221 (0.875–1.704)	0.239			1.123 (0.836–1.507)	0.441		
Others*	1.079 (0.664–1.751)	0.760			0.961 (0.621–1.487)	0.858		
Grade		0.013		0.165		0.009		0.048
III	Ref.		Ref.		Ref.		Ref.	
I	0.453 (0.201–1.019)	0.056	0.848 (0.368–1.953)	0.699	0.451 (0.223–0.910)	0.026	0.634 (0.308–1.306)	0.217
II	0.587 (0.375–0.919)	0.020	0.645 (0.409–1.019)	0.060	0.656 (0.453–0.950)	0.026	0.651 (0.446–0.951)	0.026
Stage		< 0.001		< 0.001		< 0.001		< 0.001
IIIC	Ref.		Ref.		Ref.		Ref.	
I	0.082 (0.036–0.189)	< 0.001	0.099 (0.042–0.234)	< 0.001	0.098 (0.051–0.190)	< 0.001	0.113 (0.057–0.224)	< 0.001
IIA	0.249 (0.121–0.515)	< 0.001	0.271 (0.128–0.571)	0.001	0.220 (0.121–0.398)	< 0.001	0.239 (0.129–0.441)	< 0.001
IIB	0.502 (0.240–1.047)	0.066	0.537 (0.255–1.134)	0.103	0.427 (0.233–0.783)	0.006	0.469 (0.253–0.868)	0.016
IIIA	0.816 (0.376–1.770)	0.606	0.921 (0.421–2.016)	0.837	0.583 (0.303–1.123)	0.107	0.736 (0.378–1.431)	0.366
IIIB	1.024 (0.472–2.223)	0.951	0.950 (0.433–2.084)	0.899	0.812 (0.424–1.553)	0.529	0.756 (0.391–1.462)	0.406
Subtype		0.864				0.626		
TNBC	Ref.				Ref.			
HR–/HER2 +	0.779 (0.384–1.583)	0.491			0.704 (0.374–1.327)	0.278		
HR + /HER2 +	1.130 (0.464–2.748)	0.788			0.825 (0.340–2.000)	0.670		
HR + /HER2–	0.930 (0.683–1.265)	0.642			0.900 (0.690–1.176)	0.441		
Surgery								
BCS	Ref.		Ref.		Ref.		Ref.	
Mastectomy	2.452 (1.847–3.257)	< 0.001	1.369 (1.001–1.872)	0.049	2.112 (1.666–2.676)	< 0.001	1.244 (0.956–1.619)	0.105
Radiation therapy								
No or unknown	Ref.		Ref.		Ref.		Ref.	
Yes	0.771 (0.598–0.995)	0.046	0.756 (0.566–1.010)	0.058	0.694 (0.556–0.865)	0.001	0.765 (0.596–0.983)	0.036
Chemotherapy		< 0.001		0.009		< 0.001		< 0.001
No CT	Ref.		Ref.		Ref.		Ref.	
NAC- non response	2.502 (1.533–4.081)	< 0.001	1.266 (0.744–2.155)	0.384	1.691 (1.088–2.628)	0.019	0.984 (0.610–1.587)	0.947
NAC- response	1.039 (0.700–1.544)	0.849	0.691 (0.444–1.077)	0.102	0.632 (0.440–0.907)	0.013	0.479 (0.321–0.715)	< 0.001
Adjuvant CT	0.618 (0.462–0.827)	0.001	0.658 (0.480–0.902)	0.009	0.401 (0.314–0.512)	< 0.001	0.451 (0.346–0.587)	< 0.001

*American Indian/AK native, Ascian/Pacific Islander.

**Figure 2:**
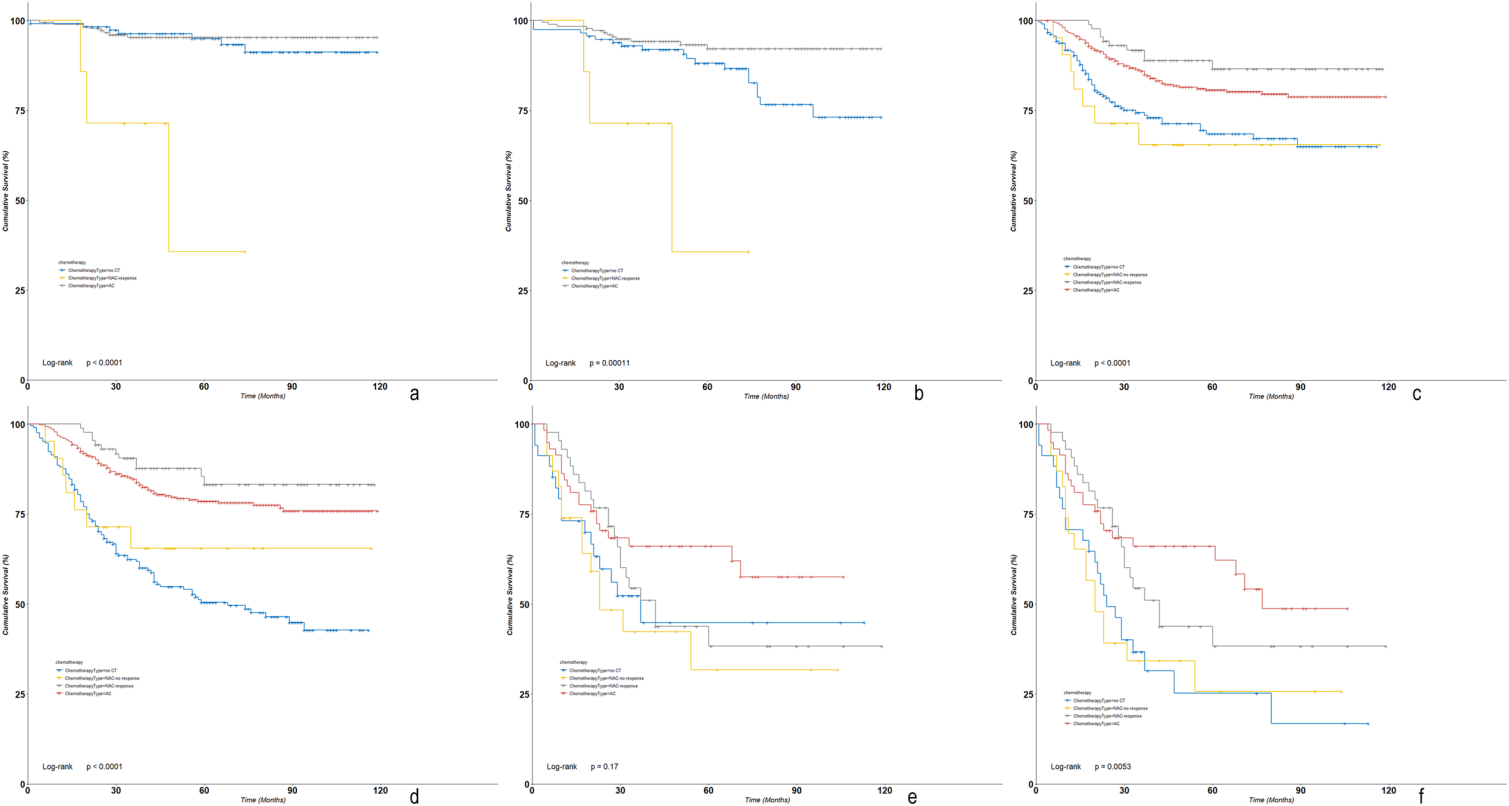
Subgroup analyses of Kaplan–Meier survival curves of BCSS and OS in MpBC based on tumor stage stratified by chemotherapy types ((**a**) KM curves of BCSS in stage I; (**b**) KM curves of OS in stage I; (**c**) KM curves of BCSS in stage II; (**d**) KM curves of OS in stage II; (**e**) KM curves of BCSS in stage III; (**f**) KM curves of OS in stage III).

There were significant differences in survival outcomes among NAC, adjuvant CT or no CT in the subgroup analyses when stratified by stage. Among MpBC patients in stage I, there were only six cases with NAC-response. Although a similar BCSS was observed between adjuvant CT and no CT (P = 0.588), those with adjuvant CT did have an improved OS (P = 0.017) (Fig. 2a,b). Among MpBC patients in stage II, those with NAC-response or adjuvant CT had a significant improved BCSS and OS compared with NAC-no response or no CT (Fig. 2c,d). Among MpBC patients in stage III, chemotherapy lost its prognostic significance as patients with NAC-response, NAC-no response, adjuvant CT and no CT had similar BCSS in most comparisons (Fig. 2e). However, chemotherapy still improved OS as patients with NAC-response and adjuvant CT had a significant or trend of improved OS compared with those with NAC-no response or no CT (Fig. 2f) (Table 4).

**Table 4:** Pairwise survival comparisons among different chemotherapy types for MpBC stratified by tumor stage.

Stage	Chemotherapy types	BCSS	OS
	Comparisons	Log rank P	Log rank P
Stage I	Adjuvant CT vs. no CT	0.588	0.017
Stage II	NAC-response vs. adjuvant CT	0.137	0.163
	NAC-response vs. NAC-no response	0.004	0.010
	NAC-response vs. no CT	0.001	< 0.001
	Adjuvant CT vs. NAC-no response	0.032	0.061
	Adjuvant CT vs. no CT	< 0.001	< 0.001
	NAC-no response vs. no CT	0.614	0.397
Stage III	NAC-response vs. adjuvant CT	0.239	0.343
	NAC-response vs. NAC-no response	0.251	0.060
	NAC-response vs. no CT	0.548	0.035
	Adjuvant CT vs. NAC-no response	0.046	0.009
	Adjuvant CT vs. no CT	0.154	0.003
	NAC-no response vs. no CT	0.623	0.989

### Survival outcomes comparisons between MpBC-TNBC and IDC-TNBC

**Figure 3:**
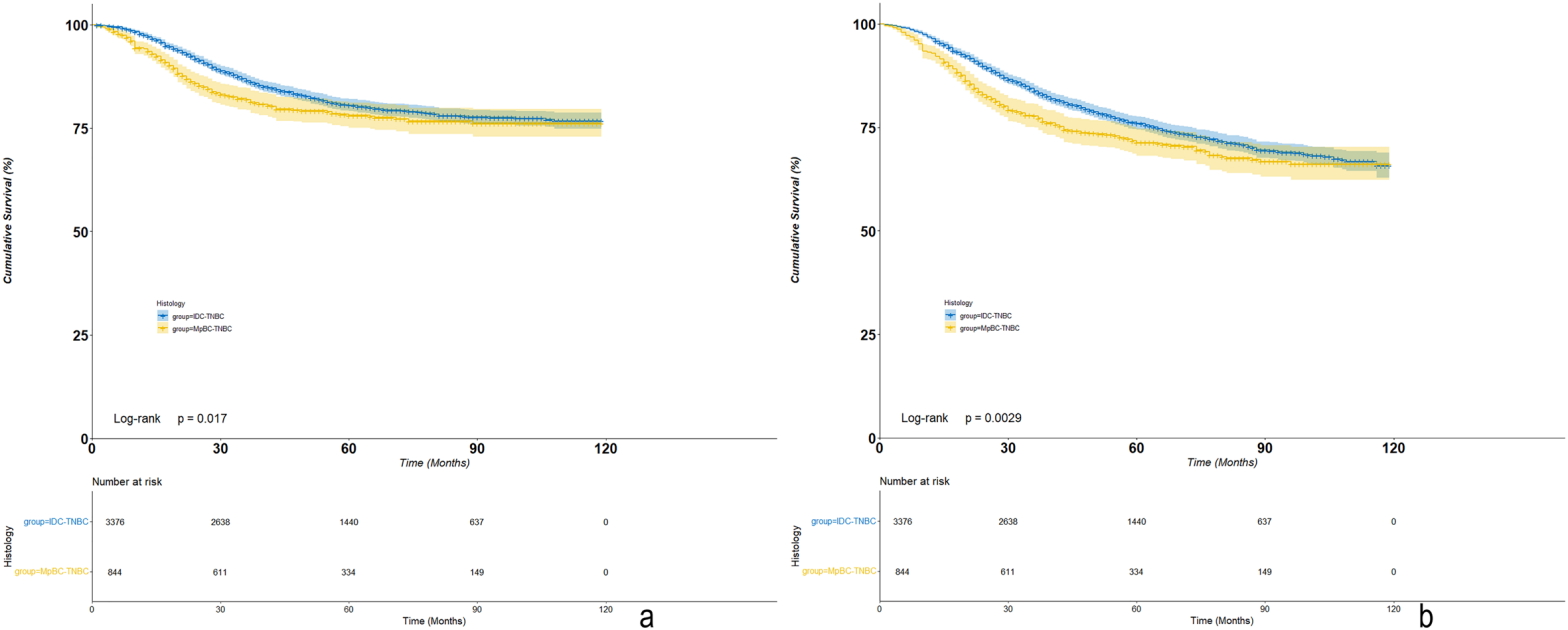
Kaplan–Meier survival curves of BCSS and OS stratified by MpBC-TNBC and IDC-TNBC ((**a**) KM curves of BCSS; (**b**) KM curves of OS).

There were 844 MpBC-TNBC cases and 21260 IDC-TNBC cases met the inclusion criteria between 2010 and 2017. A 1:4 propensity score matching (PSM) was conducted, and as a result, 844 MpBC-TNBC cases were matched with 3376 IDC-TNBC. The clinical-pathological characteristics were well-balanced between two groups after PSM (Supplementary Table). After a median 50 months (0–119 months) follow-up, IDC-TNBC had an improved BCSS (P = 0.017) and OS (P = 0.003) compared with MpBC-TNBC (Fig. 3a,b). There were statistically significant differences in BCSS (P < 0.001) and OS (P < 0.001) among chemotherapy types (NAC-response or no response, adjuvant CT or no CT) for both MpBC-TNBC and IDC-TNBC. According to COX multivariate analysis, MpBC-TNBC was an independent unfavorable prognostic factor for both BCSS (HR = 1.239 (1.046–1.468), P = 0.013) and OS (HR = 1.277 (1.104–1.477), P = 0.001) when compared with IDC-TNBC (Table 5). Meanwhile, chemotherapy was also a favorable prognostic factor for both of them. There was significant or trend of improvement for BCSS and OS among patients receiving NAC or adjuvant CT compared with no CT (Table 5).

**Table 5:** Multivariate COX regression of independent prognostic factors of MpBC-TNBC and IDC-TNBC.

	BCSS				OS			
	Univariate regression		Multivariate regression		Univariate regression		Multivariate regression	
	HR (95% CI)	P	HR (95% CI)	P	HR (95% CI)	P	HR (95% CI)	P
Histology								
IDC-TNBC	Ref.		Ref.		Ref.		Ref.	
MpBC-TNBC	1.227 (1.036–1.453)	0.018	1.239 (1.046–1.468)	0.013	1.245 (1.077–1.440)	0.003	1.277 (1.104–1.477)	0.001
Year of diagnosis	1.030 (0.999–1.063)	0.058			1.029 (1.002–1.057)	0.037	1.045 (1.017–1.075)	0.001
Age								
> 60y	Ref.				Ref.			
≤ 60y	0.970 (0.842–1.118)	0.678			0.840 (0.743–0.951)	0.006	0.914 (0.800–1.044)	0.183
Race		0.388				0.124		
White	Ref.				Ref.			
Black	1.125 (0.931–1.359)	0.223			1.124 (0.955–1.322)	0.160		
Others*	0.926 (0.684–1.253)	0.618			0.832 (0.633–1.093)	0.185		
Stage		< 0.001		< 0.001		< 0.001		< 0.001
IIIC	Ref.		Ref.		Ref.		Ref.	
I	0.086 (0.055–0.136)	< 0.001	0.102 (0.064–0.163)	< 0.001	0.119 (0.080–0.176)	< 0.001	0.131 (0.087–0.195)	< 0.001
IIA	0.188 (0.124–0.286)	< 0.001	0.217 (0.142–0.332)	< 0.001	0.212 (0.146–0.307)	< 0.001	0.228 (0.156–0.333)	< 0.001
IIB	0.422 (0.277–0.643)	< 0.001	0.458 (0.299–0.702)	< 0.001	0.405 (0.278–0.592)	< 0.001	0.431 (0.294–0.631)	< 0.001
IIIA	0.507 (0.316–0.815)	0.005	0.553 (0.342–0.894)	0.016	0.477 (0.311–0.732)	0.001	0.510 (0.331–0.788)	0.002
IIIB	0.902 (0.580–1.403)	0.648	0.885 (0.568–1.380)	0.590	0.797 (0.534–1.190)	0.267	0.767 (0.512–1.147)	0.196
Surgery								
BCS	Ref.		Ref.		Ref.		Ref.	
Mastectomy	2.094 (1.801–2.434)	< 0.001	1.333 (1.136–1.564)	< 0.001	1.815 (1.599–2.060)	< 0.001	1.268 (1.108–1.451)	0.001
Radiation therapy								
No or unknown	Ref.				Ref.			
Yes	0.979 (0.851–1.127)	0.772			0.895 (0.793–1.010)	0.073		
Chemotherapy		< 0.001		< 0.001		< 0.001		< 0.001
No CT	Ref.		Ref.		Ref.		Ref.	
NAC- non response	1.799 (1.277–2.535)	0.001	0.801 (0.564–1.139)	0.217	1.400 (1.015–1.931)	0.040	0.683 (0.488–0.956)	0.026
NAC- response	1.336 (1.070–1.668)	0.011	0.841 (0.668–1.059)	0.141	1.120 (0.918–1.365)	0.264	0.759 (0.611–0.942)	0.012
Adjuvant CT	0.686 (0.587–0.803)	< 0.001	0.702 (0.598–0.823)	< 0.001	0.634 (0.556–0.725)	< 0.001	0.656 (0.570–0.755)	< 0.001

*American Indian/AK native, Ascian/Pacific Islander.

**Figure 4:**
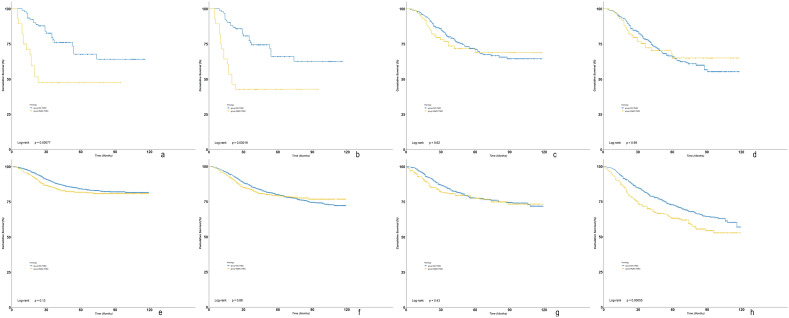
Subgroup analyses of Kaplan–Meier survival curves of BCSS and OS based on chemotherapy types stratified by MpBC-TNBC and IDC-TNBC ((**a**) KM curves of BCSS in NAC-response; (**b**) KM curves of OS in NAC-response; (**c**) KM curves of BCSS in adjuvant CT; (**d**) KM curves of OS in adjuvant CT; (**e**) KM curves of BCSS in NAC-no response; (**f**) KM curves of OS in NAC-no response; (**g**) KM curves of BCSS in no CT; (**h**) KM curves of OS in no CT.

When stratified by chemotherapy types, MpBC-TNBC and IDC-TNBC had similar survival outcomes among those with NAC-response and adjuvant CT (Fig. 4a–d). Among those with NAC-no response, IDC-TNBC had significant improved BCSS and OS compared with MpBC-TNBC (Fig. 4e,f). Among those with no CT, IDC-TNBC had a similar BCSS but an improved OS compared with MpBC-TNBC (Fig. 4g,h).

## Discussion

Evidence on treatment strategies for MpBC is limited, as management of MpBC is largely paralleled that of IDC and adopts a comprehensive therapy including surgery, chemotherapy, radiotherapy, endocrine therapy, and targeted therapy based on clinical-pathological characteristics and tumor stage. In particular, the efficacy of adjuvant chemotherapy and neoadjuvant chemotherapy is still controversial. Our study was among the largest population-based study to explore the prognostic significance of chemotherapy among MpBC receiving adjuvant CT, NAC or not any CT, and to compare the long-term survival difference between MpBC-TNBC and IDC-TNBC based on PSM.

MpBC generally has aggressive clinical and pathological features. The clinical‐pathological characteristics of the cohort of MpBCs in the current study was in line with those reported in the literature^1,2,7,9–14^, in which most MpBCs were presented in larger tumor, higher histologic grade, higher number of positive lymph nodes and majority of TN phenotype. In this study, 82.5% MpBC cases had histologic grade III, 19.6% had positive lymph nodes, 21.2% had T3 or T4 disease, and 13.3% were in stage III. Besides, 71.2% cases were in TN phenotype, which was consistent with previous studies^2,14^. The rate of HER2 overexpression (5.7%) and positive hormone receptor (HR) status (24.9%) was in accord with previous reports^15,16^. However, HR and HER2 status remained no impact on prognosis of MpBC. In patients with or without HER2 overexpression, the prognosis of single HR + tumor was similar to single HR + or double HR- tumor^15^. The role of HER2 in MpBC patients remains unclear^16,17^. Based on multivariate analysis in this study, molecular subtype (TN as reference) was not an independent prognostic factor of BCSS or OS for MpBC.

In spite of high proportion of aggressive characteristics, the effectiveness of standard chemotherapy regimens for MpBC was controversial, as in most studies MpBC was considered in part chemo-resistant^18–20^. The poor response to anthracyclines and taxanes suggested chemoresistance probably associated to epithelial-mesenchymal transition (EMT)^5,10,21^ which was frequently observed upregulated in these tumors^22–24^. Despite the traditional notion that MpBC is resistant to chemotherapy, systemic chemotherapy is administered to 53.4–73.1% MpBC patients^4,25^. A recent study by Ong et al. reviewed 2500 patients with MpBC and found that chemotherapy use versus no chemotherapy was significantly associated with improved survival, although the specific chemotherapy regimens utilized were not reported^2^. Several studies also conducted prognostic nomograms for predicting the OS for MpBC, in which chemotherapy was a favorable prognostic factor^26–28^.

The role of chemotherapy in MpBC has been confirmed in this study, and the potential subgroups benefiting from CT was also explored. MpBC patients who received adjuvant CT and NAC with response had an improved BCSS and OS compared with those without CT. Due to limited cases, patients with NAC-response only had an obvious trend of BCSS improvement. However, the HR value in the multivariate analysis was similar to that of adjuvant CT group, indicating that it reduced the death risk to the same extent. Among patients in stage I, those with adjuvant CT did not show significant survival benefit compared with those without CT. It could be postulated that surgery still remained to be the standard therapy in most early-stage MpBC case such as stage I, which had a favorable prognosis and a radical surgery might be adequate for cure with systemic therapy exempt safely. Likewise, according to Chen’s study, among node-negative MpBC, CT improved the prognosis of T1c MpBC patients but not T1a and T1b patients to a beneficial extent^29^. Meanwhile, among locally advanced disease such as stage III, patients with adjuvant CT, no CT, NAC with or without response had similar BCSS in most cases. However, CT showed OS improvement compared with no CT or NAC-no response in stage I and stage III. It was suggested that when MpBC progressed to an advanced stage, CT might have limited benefit for significant survival improvement. Perhaps the limited cases in stage III might restrict the statistical efficacy to tell the difference. Several studies have reported that the effect of CT associated with better outcome was limited in early-stage cases^21,30^. However, only among stage II disease for which systemic therapy was essential, patients with adjuvant CT or NAC with response had better prognosis than those without CT or receiving NAC without response. It could be postulated from our study that chemotherapy should be included as the multi-disciplinary treatment for MpBC patients with high-risk features, and early screening was also of first-priority for MpBC.

One of the strengths of this study was that it distinguished the response to NAC to explore respectively the significance of NAC for MpBC. Although the response to NAC can predict clinical outcome, there is a dearth of studies evaluating response to NAC in MpBC. In this study, 15.3% MpBC patients received NAC while 54.6% received adjuvant CT. A study from the European Institute of Oncology revealed that just 7.8% of MpBC received NAC and the majority undergoing adjuvant CT^31^. An earlier NCDB study demonstrated that NAC was used in only 15.5% of patients with MpBC^1^. MpBC has been considered poorly responsive to NAC. Previous small case series demonstrated pathological complete response (pCR) rates of approximately 10%, substantially lower than that of classic IDC^32,33^. As a result, some argued that MpBC should not receive NAC^31,32^. In this study, only 12.2% MpBC patients receiving NAC achieved CR while 75.7% showed response to NAC. According to multivariate analysis, NAC-response showed an obvious improvement for BCSS and OS, just like adjuvant CT. However, NAC-no response could not improve survival outcomes. Based on Haque’s study, there was significantly improved 5-year OS among MpBC patients with pCR^34^. It suggested that CT had important prognostic significance for MpBC and the response to NAC could help select favorable subsets which may experience long-term favorable prognosis^14,35,36^. Further researches are warranted to explore biomarkers to ensure appropriate patient selection^37^.

Although the majority of MpBC is presented with TN phenotype, the survival difference between MpBC-TNBC and IDC-TNBC is still controversial. Many retrospective studies with small sample size agreed that the prognosis of MpBC-TNBC was significantly worse than that of IDC-TNBC^2,3,7,13^, while other research indicated that these two had similar overall and disease‐free survival^31,38^. Larger studies documented a significant worse prognosis of MpBC-TNBC than other IDC-TNBC from the NCDB database, and the significant survival difference was maintained at multivariable analysis. As MpBC tended to present with more locally advanced disease in comparison to IDC-TNBC^20^, PSM was adopted to balance the baseline differences in this study. Yet MpBC was confirmed as an independent unfavorable prognostic factor compared with IDC-TNBC based on multivariate COX regression after a successful PSM. Furthermore, chemotherapy was also a favorable prognostic factor for BCSS and OS among MpBC-TNBC and IDC-TNBC based on the multivariate analysis in this study. Subgroup analysis indicated that MpBC-TNBC had similar survival outcomes compared with IDC-TNBC when they received adjuvant CT or NAC with response. It suggested that chemotherapy was of most importance to these two aggressive subtypes. The current standard of care for MpBC follows the same guidelines as IDC-TNBC. According to Polamraju’s study, CT was associated with improved OS among MpBC and IDC-TNBC^3^. On the contrary, IDC-TNBC had significant improved BCSS and OS compared with MpBC-TNBC when they receiving NAC but with no response, and it still had an improved OS compared with MpBC-TNBC when they did not receive CT. It further suggested that the histology of MpBC might confer an additional survival disadvantage. Mutations in *PIK3CA, PIK3R1, ARID1A, FAT1*, and *PTEN* were more frequently harbored in MpBC in comparison to IDC-TNBC, which may contribute to the poor clinical outcomes in MpBC^39,40^ and warrant further research.

The strengths of this study were obvious, such as large sample size, classification of chemotherapy types of NAC-response, NAC-no response, adjuvant CT and no CT in all analyses, and comparison with IDC-TNBC based on PSM. However, some limitations should also be addressed. Firstly, although chemotherapy was confirmed of great significance to MpBC, the chemotherapy regimens, duration and response was unavailable in the SEER database. Secondly, MpBC has been shown to be extremely heterogeneous in morphology and in survival outcomes^17,41^. However, in this study, all MpBC cases together with the special subtypes were included, and chemotherapy was confirmed as an independent favorable prognostic factor for BCSS and OS. Lastly, the intrinsic bias could not be avoided in spite of the large sample size.

In conclusion, chemotherapy was of important significance to the prognosis of MpBC and should be integrated in the comprehensive treatment for MpBC. Further researches are warranted to explore the potential biomarkers in MpBC to predict response to chemotherapy.

## Methods

### Patient cohort and stratification

**Figure 5:**
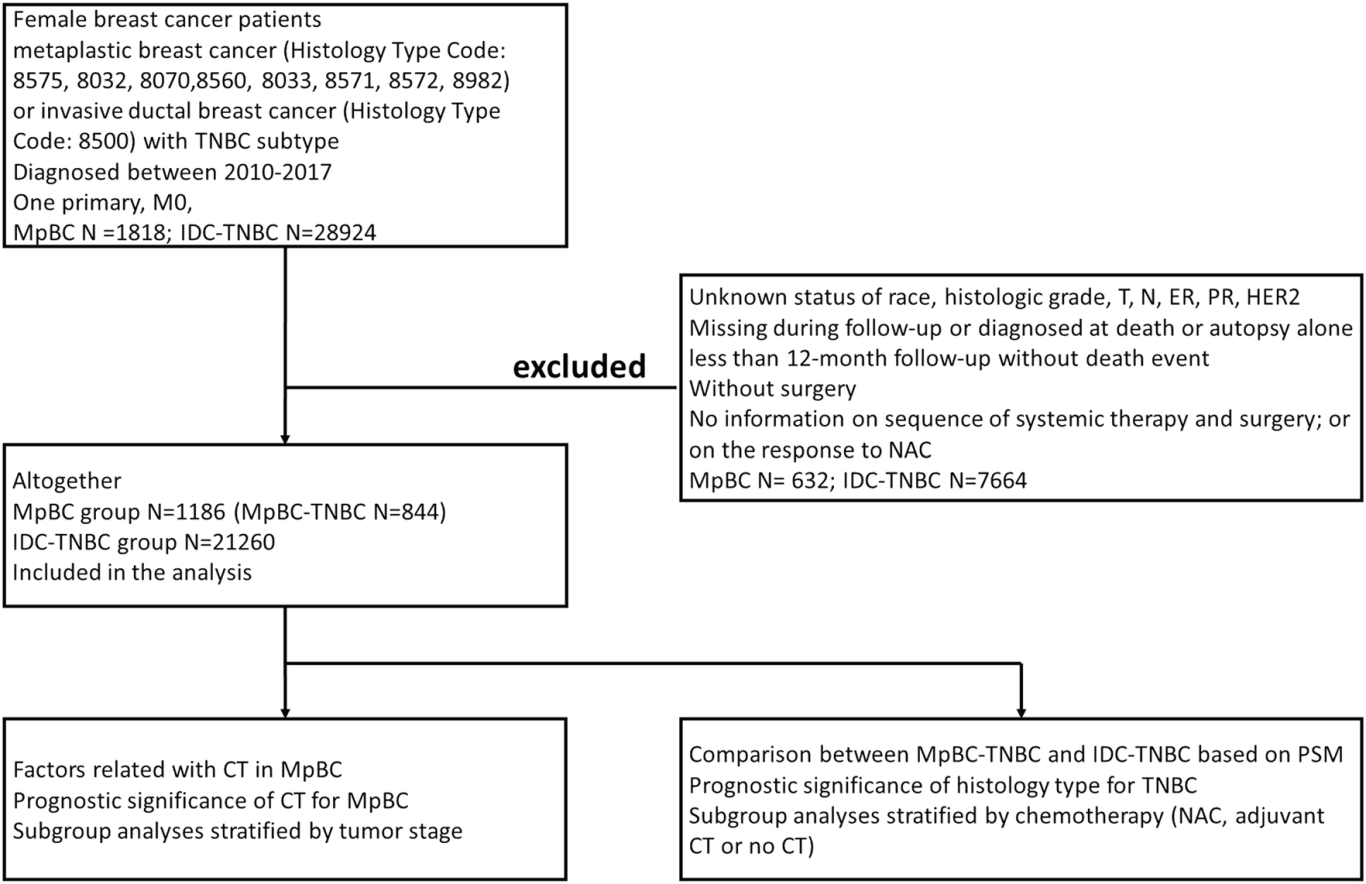
The diagram of patient cohort selection process and the overview of the study consort.

The patient population in this study used data derived from the Surveillance, Epidemiology, and End Results (SEER) database released in 2021. Female unilateral primary MpBC of no special type (MpBC-NST) (coded as 8575) between 2010 and 2017 were enrolled. Besides, some special subtypes of MpBC were also collected, that was, spindle cell carcinoma (coded as 8032), squamous cell carcinoma (8070), low-grade adenosquamous carcinoma (8560), sarcomatoid carcinoma (8033), MpBC with chondroid differentiation (8571), fibromatosis-like MpBC (8572) and myoepithelial carcinoma (8982). Invasive ductal breast cancer with triple negative subtype (IDC-TNBC) which met the inclusion criteria above were also enrolled for comparison with MpBC-TNBC. Patients who had more than one primary cancer, metastasis disease at diagnosis or no surgery performed or no record of surgery, who were diagnosed at death or autopsy alone, missing during follow up or less than 12 months follow-up without death event were excluded. Patients with unknown race, histologic grade, T or N category, ER or PR or HER2 status were also excluded. Histologic grade III was defined as poorly differentiated and anaplastic histologic grades disease. CT status ‘yes’ together with response information to neoadjuvant therapy was defined as neoadjuvant CT (NAC), among which response to NAC stated as ‘complete response’, ‘partial response’ and ‘response to treatment, but not noted if complete or partial’ was defined as ‘NAC-response’, while ‘no response’ was defined as ‘NAC-no response’. CT status ‘yes’ together with ‘systemic therapy after surgery’ was defined as adjuvant CT. CT status ‘no or unknown’ together with no systemic therapy was defined as ‘no CT’. The patient cohort selection process and study consort diagram were shown in Fig. 5.

We had the permission to SEER data access. As SEER database is an open public database without involving personal information, our institution review board (IRB) has determined that no ethical approval is required.

### Statistical analysis

The proportions of clinical-pathological characteristics of MpBC stratified by NAC, adjuvant CT or no CT were compared by means of Pearson’s Chi square. The follow-up was calculated till 31 December 2019. Breast cancer-specific survival (BCSS) was defined as the interval from breast cancer diagnosis to death from breast cancer or the last follow-up. Overall survival (OS) was defined as the interval from diagnosis to death from any cause or the last follow-up. The Kaplan–Meier method was used to construct survival curves, and the log-rank test was used to estimate the differences in survival outcomes between groups. Significant independent prognostic factors were evaluated by means of Cox hazards model in the format of adjusted hazard ratios (HRs) with 95% confidence intervals (CIs). In order to overcome the effects of baseline differences on survival outcomes in the MpBC-TNBC and IDC-TNBC groups, PSM method was adopted with factors such as diagnosis year stage, age, race, tumor stage, breast surgery, chemotherapy types and radiation therapy enrolled. All the statistical tests were two sided, and statistical significance was defined as P value less than 0.05. SPSS 22.0 and R statistics 4.2.2 were used for statistical calculations. 

### Ethics declarations and consent to participate

All procedures performed in studies involving human participants were in accordance with the ethical standards of the institutional and/or national research committee and with the 1964 Helsinki declaration and its later amendments or comparable ethical standards. This article does not contain any studies with animals performed by any of the authors. As SEER database is an open public database without involving personal information, informed consent was consequently not required. The Obstetrics and Gynecology Hospital of Fudan University IRB has reviewed the project and has determined this project does not meet the definition of human subject research under the purview of the IRB according to the national regulations.

### Supplementary Information

Supplementary Tables.

## Data Availability

Publicly available datasets were analyzed in this study. The data can be found here: https://seer.cancer.gov/data/.
