# Supplementary material for: Chemotherapy is of prognostic significance to metaplastic breast cancer
Source: Sci Rep. 2024 Jan 12;14:1210. doi: 10.1038/s41598-024-51627-1 (PMC10786888; doi:10.1038/s41598-024-51627-1)
Supplement: Supplementary file 1 — Supplementary Tables. [file 41598_2024_51627_MOESM1_ESM.docx]

Supplementary Table balanced clinical-pathological characteristics between MpBC-TNBC and IDC-TNBC

|  | **MpBC-TNBC** | |  | **IDC-TNBC** | |  | **P** |
| --- | --- | --- | --- | --- | --- | --- | --- |
|  | *No.* | *%* |  | *No.* | *%* |  |  |
| **Year of diagnosis** |  |  |  |  |  |  | 0.952 |
| 2010-2011 | 193 | 22.9% |  | 749 | 22.2% |  |  |
| 2012-2013 | 187 | 22.0% |  | 742 | 22.2% |  |  |
| 2014-2015 | 214 | 25.4% |  | 885 | 26.2% |  |  |
| 2016-2017 | 250 | 29.6% |  | 1000 | 29.6% |  |  |
| **Age** |  |  |  |  |  |  | 0.975 |
| ≤60 | 373 | 44.2% |  | 1490 | 44.1% |  |  |
| >60 | 471 | 55.8% |  | 1886 | 55.9% |  |  |
| **Race** |  |  |  |  |  |  | 0.865 |
| White | 662 | 78.4% |  | 2646 | 78.4% |  |  |
| Black | 128 | 15.2% |  | 528 | 15.6% |  |  |
| Others* | 54 | 6.4% |  | 202 | 6.0% |  |  |
| **Stage** |  |  |  |  |  |  | 0.994 |
| I | 214 | 25.4% |  | 869 | 25.7% |  |  |
| IIA | 377 | 44.7% |  | 1508 | 44.7% |  |  |
| IIB | 155 | 18.4% |  | 624 | 18.5% |  |  |
| IIIA | 41 | 4.9% |  | 147 | 4.4% |  |  |
| IIIB | 47 | 5.6% |  | 190 | 5.6% |  |  |
| IIIC | 10 | 1.2% |  | 38 | 1.1% |  |  |
| **Surgery** |  |  |  |  |  |  | 0.161 |
| BCS | 387 | 45.9% |  | 1639 | 48.5% |  |  |
| mastectomy | 457 | 54.1% |  | 1737 | 51.5% |  |  |
| **Chemotherapy Type** |  |  |  |  |  |  | 0.802 |
| NAC-no response | 28 | 3.3% |  | 92 | 2.7% |  |  |
| NAC-response | 84 | 10.0% |  | 326 | 9.7% |  |  |
| Adjuvant CT | 460 | 54.5% |  | 1847 | 54.7% |  |  |
| no CT | 272 | 32.2% |  | 1111 | 32.9% |  |  |
| **Radiation therapy** |  |  |  |  |  |  | 0.963 |
| yes | 414 | 49.1% |  | 1653 | 49.0% |  |  |
| no or unknown | 430 | 50.9% |  | 1723 | 51.0% |  |  |
